# Comparative Phytochemical Analysis of *Gastrodiae Rhizoma* Peel and Core and Their Lifespan-Extending Potential in *Caenorhabditis elegans*

**DOI:** 10.3390/molecules30173474

**Published:** 2025-08-23

**Authors:** Baoshan Li, Ke Mo, Lipeng Zhou, Yanjun Wang, Yaping Li, Wei Zhang, Chenghao Zhu, Zhirong Sun

**Affiliations:** 1 School of Chinese Materia Medica, Beijing University of Chinese Medicine, Beijing 102488, China; 20230935262@bucm.edu.cn (B.L.); moke2020@126.com (K.M.); 20240941476@bucm.edu.cn (L.Z.); 20220935153@bucm.edu.cn (Y.W.); 20240935178@bucm.edu.cn (Y.L.); 2 Ningqiang County Traditional Chinese Medicinal Industry Development Center, Hanzhong 724400, China; 15523840536@163.com; 3 Guangxi Institute of Botany, Chinese Academy of Sciences, Guilin 541006, China

**Keywords:** *Gastrodia elata* Blume, chemical constituents, GC–IMS, UHPLC–MS, relative odor activity value, *Caenorhabditis elegans*

## Abstract

*Gastrodiae Rhizoma* (tianma, TM), a traditional medicine that has food and medicine homology, faces controversy over retaining its epidermis (tianma pi, TP) during processing due to unclear phytochemical value. This study presents the first integrated approach combining GC–IMS, UHPLC–MS, and *Caenorhabditis elegans* (*C. elegans*) aging models to compare TP with the tuber core (tianma xin, TX). The results include the following: (1) A total of forty-seven volatile compounds were identified by GC–IMS, including 12 key aroma substances via relative odor activity value (ROAV ≥ 1), of which seven ((*Z*)-4-heptenal, β-citronellol, hexanal, 1-pentanol, 1-octen-3-one, 2-methylpropanol, and 2-butanone) were enriched in TP. (2) Non-targeted metabolomics revealed 1025 metabolites via UHPLC–MS, highlighting phenylpropanoid biosynthesis as the primary differential pathway (*p* < 0.05). Phenylpropanoids and polyketides exhibited predominant enrichment in TP (|log2FC| > 2, VIP > 1, *p* < 0.01). (3) In *C. elegans* models, TP outperformed TX in pharyngeal pumping (4.16%, *p* < 0.05), while both extended stress-resistant lifespan (*p* < 0.01). In conclusion, TP plays an essential role in establishing the characteristic odor profile of TM and retaining bioactive components, particularly phenylpropanoids. Preserving TP during processing optimally maintains the distinctive aroma profile and pharmacological value of TM, which provides valuable guidance for industrial utilization.

## 1. Introduction

*Gastrodiae Rhizoma* (tianma, TM), the dried tuber of the orchid species *Gastrodia elata* Blume (Orchidaceae), has served as a food resource with remarkable nutritional value in China for over 2000 years [1,2]. Renowned for its distinctive flavor profiles and exceptional nutraceutical efficacy, traditional medicinal cuisine, including “Tianma stewed black chicken”, “Tianma sleeping porridge” and “Tianma boiled pig brain”, has been extensively embraced and perpetuated within the folk culture [1,2]. Contemporary pharmacological investigations have revealed its multifaceted bioactivities, including neuroprotective activities [3,4,5], regulation of cardiovascular and cerebrovascular systems [6,7,8], intestinal protection effect [9,10], anti-cancer activities [11], antioxidant and antiaging effects [12,13], among others. Notably, TM was officially designated as a novel food resource by China’s National Health Commission in 2023 [14]. Nevertheless, the processing methods for TM remain controversial. Historical records reveal discrepancies [15,16]: peeling protocols were notably absent in the Newly Revised Materia Medica (Tang Dynasty) and Kaibao Materia Medica (Song Dynasty), while the Illustrated Materia Medica (Song) and Compendium of Materia Medica Essentials (Ming) documented peeled rhizomes for raw consumption or candied preparation. The mainstream processing of TM from 1911 to 1985 mandated epidermal removal prior to dehydration [15]. Although the current Chinese Pharmacopoeia advocates retaining the tuber peel (tianma pi, TP), our literature review and field investigations revealed that peeling practices persist in both folk culinary applications and scientific research [17,18]. The controversy surrounding TM processing fundamentally lies in insufficient scientific understanding of TP.

The developmental regulation of plant metabolite accumulation drives tissue-specific metabolic variations [19,20]. Comparative phytochemical analyses revealed marked differential distribution of bioactive compounds between TP and TX (tianma xin, TX) [21,22]. The UPLC-Q-Exactive MS, recognized for its superior chromatographic resolution and mass accuracy, has become an excellent method to profile plant-specialized metabolites [23,24]. Therefore, this study employed UPLC-Q-Exactive MS to conduct an in-depth analysis of metabolites between TP and TX. In addition, *Gastrodia elata* Blume derives nutrients through symbiotic interactions with *Armillaria* spp., during which its tuber develops a distinctive “horse-urine odor” [25]. Notably, this odor intensity has been empirically validated as a critical quality indicator [26]. Although studies confirm its multicomponent volatile composition [25], the principal origin of these volatile components remains unelucidated. Gas chromatography–ion mobility spectrometry (GC–IMS), an analytical technique recognized for operational efficiency and trace-level detection capabilities [27], represents a robust platform to unravel a such distinctive aroma signature. The relative odor activity value (ROAV) was employed to quantify the contribution of individual compounds to the overall odor profile.

United Nations demographic projections indicate that the global population aged ≥ 65 years may reach 16% by 2050, suggesting age-related health challenges will likely persist as a priority in public health discourse [28]. Intriguingly, phytochemical investigations have revealed geroprotective potential in multiple TM constituents. For instance, gastrodin has been shown to prolong lifespan via redox homeostasis modulation and ameliorate neurodegeneration in Pink1 (B9) Parkinson’s disease models [29]. Similarly, parishins demonstrated cardioprotective effects against age-related decline in murine systems, potentially through Cyp2e1 suppression and SIRT1 pathway activation [30]. Additionally, adenosine derivative T1-11 alleviated aging in mice through mitigation of oxidative damage, achieved by restoring antioxidant enzyme activities (CAT/SOD/GSH-Px) and reducing cortical–hippocampal malondialdehyde (MDA) levels [12,31]. The *Caenorhabditis elegans* (*C. elegans*) shares over 65% of genes associated with human diseases [32] and is recognized as a classic model organism for aging research due to its short lifespan, high reproductive capacity, and ease of laboratory cultivation [33,34,35,36]. Furthermore, *C. elegans* exhibits age-associated degenerative alterations in functional characteristics of reproductive, locomotor, neural, and metabolic systems, which parallel human senescence processes [36]. Based on these considerations, this investigation employed the *C. elegans* model for comparative analysis of TP and TX.

In summary, this study employed UPLC-Q-Exactive MS and GC–IMS to conduct a comparative analysis of chemical constituents in the TP and TX, as well as utilizing the *C. elegans* model to evaluate their relative lifespan-extending efficacy. To the best of our knowledge, this represents the first comprehensive comparative investigation of the TP and TX, aiming to facilitate rational utilization of TM’s homologous food–medicine resources.

## 2. Results

### 2.1. HPLC Analysis

**Figure 1:**
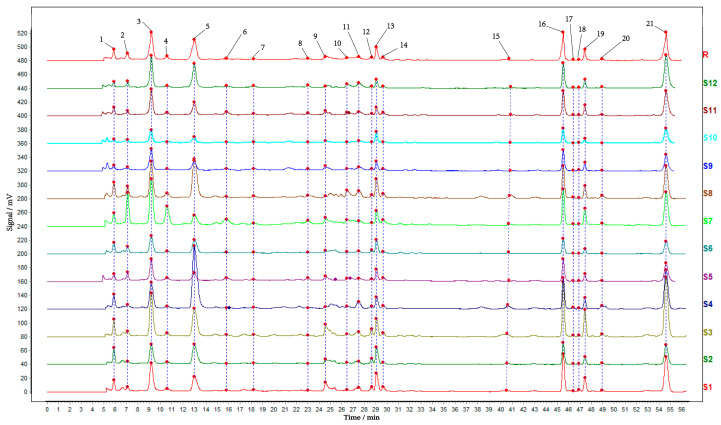
HPLC chromatograms of *Gastrodiae Rhizoma* and its peel and core. Note: 1. The S1–S4 were the whole TM, the S5–S8 were the TX, and the S9–S12 were the TP. The R was a control map generated by software. Peaks 1, 3, 5, 13, 16, 19 and 21 were adenosine, gastrodin, p-hydroxybenzyl alcohol, and parishin E, B, C, and A, respectively. 2. “TM” refers to the whole of *Gastrodiae Rhizoma*, “TP” denotes the peel of *Gastrodiae Rhizoma*, and “TX” represents the core of *Gastrodiae Rhizoma*.

HPLC was performed to explore the chemical composition differences among different parts of TM. The chromatographic data were imported into the “TCM Chromatographic Fingerprint Similarity Evaluation System” to obtain similarity analysis. The analysis results in Table 1 showed that the similarity was greater than 0.867, which proved that the chemical composition of all samples was roughly similar. After matching, a total of 21 common peaks were obtained, with the seven predominant constituents unambiguously identified as adenosine (peak 1), gastrodin (peak 3), p-hydroxybenzyl alcohol (peak 5), and parishins E (peak 13), B (peak 16), C (peak 19), and A (peak 21) through comparative analysis with authenticated reference standards (Figure 1).

### 2.2. GC–IMS Analysis

**Figure 2:**
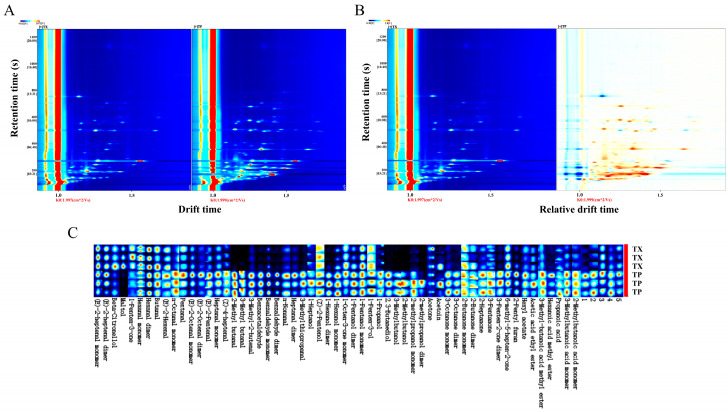
GC–IMS analysis of different parts of *Gastrodiae Rhizoma* ((**A**): Two-dimensional spectrum of volatile components by GC-IMS; (**B**): Differential spectrogram of volatile components by GC-IMS; (**C**): Fingerprint spectrum of volatile components). Note: “TP” denotes the peel of *Gastrodiae Rhizoma*, while “TX” represents the core of *Gastrodiae Rhizoma*.

**Figure 3:**
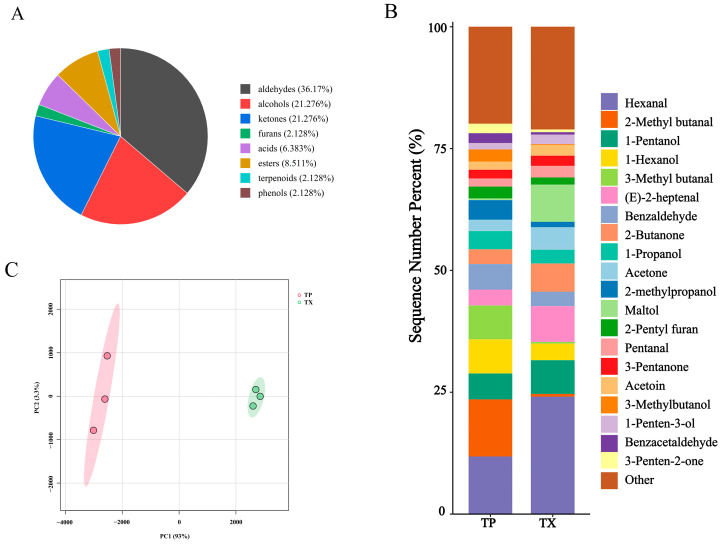
Component analysis of volatile compounds in different parts of *Gastrodiae Rhizoma* ((**A**): Pie chart of volatile component classification; (**B**): Cumulative distribution of volatile components in different parts of TM; (**C**): PCA). Note: “TP” denotes the peel of *Gastrodiae Rhizoma*, while “TX” represents the core of *Gastrodiae Rhizoma*.

**Figure 4:**
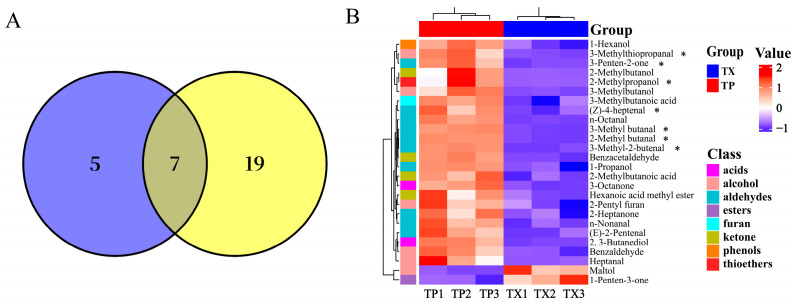
Venn diagram and cluster heat map of differential volatile compounds in different parts of *Gastrodiae Rhizoma* ((**A**): Venn diagram of key odor components and differential compounds; (**B**): Cluster heat map of differential volatile compounds in different parts of *Gastrodiae Rhizoma*, and “*” indicated the key odor components among the differential compounds.). Note: “TP” denotes the peel of *Gastrodiae Rhizoma*, while “TX” represents the core of *Gastrodiae Rhizoma*.

Differences in volatile organic compounds (VOCs) between TP and TX were investigated using gas chromatography–ion mobility spectrometry (GC–IMS). The method revealed that certain individual compounds may generate multiple signals or spots (monomers or dimers) due to concentration-dependent behavior. A total of 52 distinct VOCs were identified, comprising 17 aldehydes, 10 alcohols, 10 ketones, 4 esters, 3 acids, 1 furan, 1 terpenoid, 1 phenol, and 5 unidentified compounds (Figure 2 and Figure 3A). The stacked bar chart of relative peak areas (Figure 2B) illustrates distinct compositional profiles: TP is predominantly characterized by hexanal, 2-methylbutanal, 1-pentanol, 1-hexanol, 3-methylbutanal, and benzaldehyde, whereas TX exhibits higher abundances of hexanal, 1-pentanol, maltol, (*E*)-2-heptenal, 2-butanone, and acetone. Volatile compound fingerprinting, generated by the Gallery-plot plugin of VOCal data processing software (0.4.03, G.A.S. Dortmund, Germany), visually highlights significant compositional divergence between TP and TX (Figure 2C). Principal component analysis (PCA) of the 47 identified compounds corroborated these distinctions (Figure 3C). To further elucidate these variations, a clustering heatmap was constructed based on 26 differential compounds (fold-change > 2.0, variable importance in projection (VIP) > 1.0, *p* < 0.05) identified between the TP and TX (Figure 4B and Supplementary Figure S1). Notably, twenty-four of the twenty-six differential compounds were significantly enriched in TP, while TX showed exclusive enrichment of only two compounds (maltol and 1-penten-3-one). Collectively, TP demonstrates marked superiority in volatile compound diversity and abundance.

To elucidate key odorants in TP and TX, we performed ROAV analysis on GC–IMS-detected VOCs, identifying critical odor constituents. Compounds with ROAV ≥ 1 were designated as key odorants. Based on ROAV analysis, the characteristic aroma of TP arises from synergistic interactions among multiple volatile components (Table 2 and Supplementary Table S5): (*Z*)-4-heptenal (grassy and oily), β-citronellol (sweet rosy), hexanal (fresh-green-fatty-fruity), 1-pentanol (balsamic), 2-methylpropanol (fresh-alcoholic-leather), 1-octen-3-one (strong earthy-mushroom-vegetable-fishy composite), 2-butanone (fruity-camphoraceous), 3-methyl-2-butenal (fruity), 3-methylthiopropanal (onion-meat-fruity complex), 3-methyl butanal (chocolate-fatty), 2-methyl butanal (almond-cocoa-malt), and 3-penten-2-one (fruity), which collectively establish the distinctive sensory profile. Notably, 3-penten-2-one undergoes significant aromatic transformation during storage, with its initial fruity character progressively transitioning to spicy characteristics. This dynamic transition provides a critical reference value for determining the optimal medicinal material storage period. Among these compounds, only the first seven ((*Z*)-4-heptenal, β-citronellol, hexanal, 1-pentanol, 1-octen-3-one, 2-methylpropanol, and 2-butanone) were identified as key odorants in TX. The Venn diagram analysis identified seven differential odor compounds between TP and TX, which were annotated with asterisks in the corresponding heatmap (Figure 4A,B). Furthermore, the hierarchical clustering heatmap visually demonstrates that all these differential compounds were enriched in TP.

### 2.3. Metabolomics Analysis

**Figure 5:**
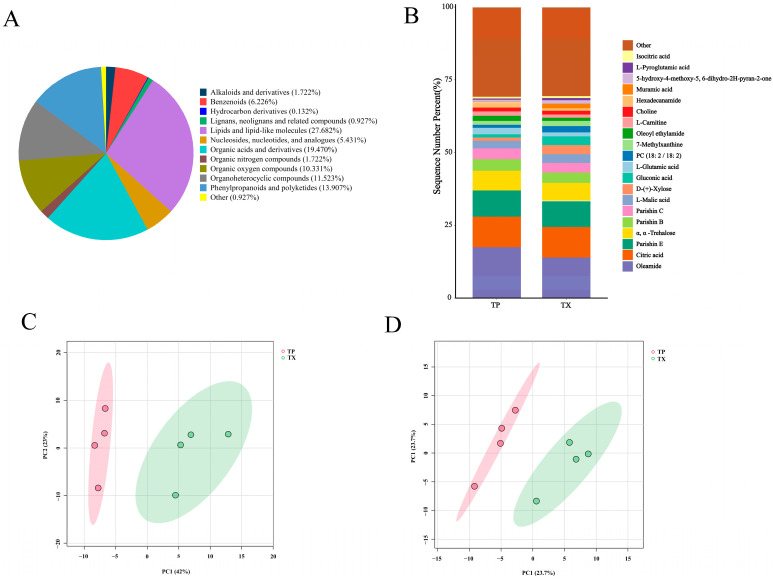
Component analysis of metabolites in different parts of *Gastrodiae Rhizoma* ((**A**): Pie chart of metabolites classification; (**B**): Cumulative distribution of metabolites in different parts of *Gastrodiae Rhizoma*; (**C**): PCA in positive ion mode; (**D**): PCA in negative ion mode). Note: “TP” denotes the peel of *Gastrodiae Rhizoma*, while “TX” represents the core of *Gastrodiae Rhizoma*.

**Figure 6:**
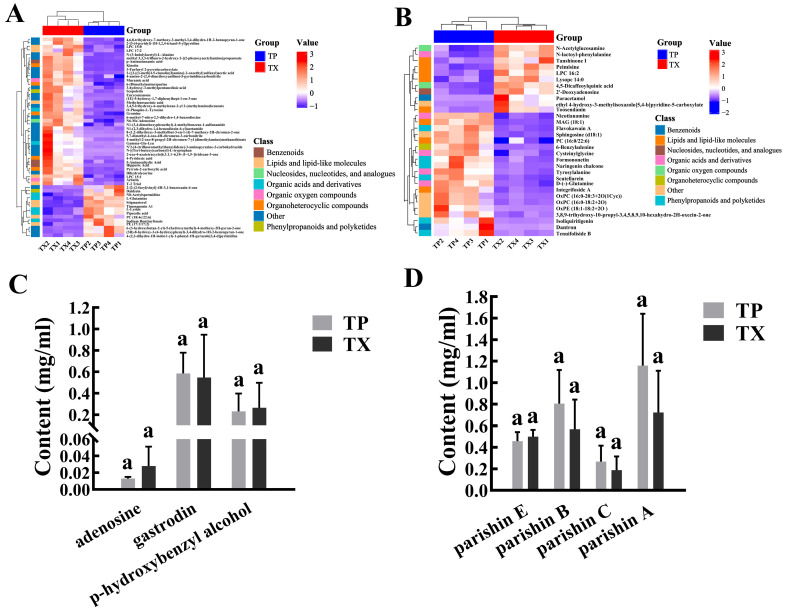
Cluster heat map of differential metabolites and quantitative analysis of the main active ingredients in different parts of *Gastrodiae Rhizoma* ((**A**): Cluster heat map in positive ion mode; (**B**): Cluster heat map in negative ion mode; (**C**,**D**): Quantitative analysis of the main active ingredients). Note: 1. “TP” denotes the peel of *Gastrodiae Rhizoma*, while “TX” represents the core of *Gastrodiae Rhizoma*. 2. The same letter in the figure indicates that the difference is not significant (*p* > 0.05).

To further investigate the distribution of specialized metabolites and characterize differential metabolites (DMs) between TP and TX, plant metabolomics analysis was conducted using LC-MS. A total of 1025 metabolites were identified in TP and TX through standard identification procedures, comprising 615 in positive ion mode and 410 in negative ion mode. Further details on these metabolites are provided in Supplementary Tables S6–S9. The identified metabolites primarily comprised the following categories: lipids and lipid-like molecules; phenylpropanoids and polyketides; organic acids and derivatives; organic oxygen compounds; organoheterocyclic compounds; nucleosides, nucleotides, and analogues; benzenoids; lignans, neolignans, and related compounds; alkaloids and derivatives; and organic nitrogen compounds (Figure 5A). A stacked bar chart was subsequently generated based on the relative peak areas of individual metabolites (Figure 5B). As illustrated in Figure 6B, oleamide, citric acid, parishin E, α,α-trehalose, parishin C, and DL-malic acid constitute the six most abundant components in TP and TX. While TP and TX demonstrated compositional similarities consistent with previous findings in other plant species [43], discernible disparities in metabolite profiles, evident from the stacked chromatograms, suggest specific differentiation in specialized metabolic pathways.

In the PCA of these metabolites (615 in positive ion mode and 410 in negative ion mode), five principal components (PCs) collectively explained 92.5% of the variance in the samples in the positive ion mode and 91.7% of the variance in the samples in the negative ion mode, respectively. The scatter plots of PCA scores revealed a notable distinction between TP and TX (Figure 5C,D). The confidence interval used was 95% by default. Metabolite data were analyzed using orthogonal partial least squares–discriminant analysis (OPLS–DA) and fold change (FC) analysis to further identify DMs between groups (Supplementary Figure S2). A total of 85 DMs were identified based on the criteria of |log2FC| > 2.0, variable importance in projection (VIP) > 1.0, and *p* < 0.01. Among these, 54 DMs were detected in positive ion mode and 31 in negative ion mode (Supplementary Tables S10 and S11).

The clustered heatmaps of DMs are presented in Figure 6A,B. These DMs were predominantly enriched in phenylpropanoids, lipids, nucleic acids, oxygen-containing acids, and organic oxygen compounds. Specifically, the TP exhibited higher abundances of organic acids and derivatives and phenylpropanoids and polyketides, whereas the TX contained more organoheterocyclic compounds, organic oxygen compounds, and lipids and lipid-like molecules. Notably, the differential metabolites did not include established primary active constituents of TM, such as adenosine, gastrodin, p-hydroxybenzyl alcohol, parishin E, parishin B, parishin C, and parishin A. To further corroborate this finding, we conducted quantitative HPLC analysis of these components in TP and TX. Results demonstrated no statistically significant differences (*p* > 0.05, *t*-test) in the concentrations of these compounds between TP and TX, suggesting that TP possesses bioactive potential comparable to TX.

**Figure 7:**
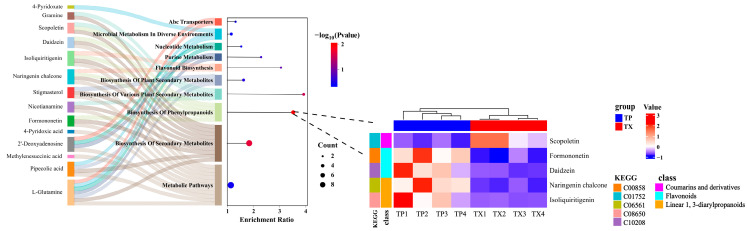
Sankey bubble plots of KEGG metabolic pathways, and the heatmap illustrates the distribution of metabolites enriched in the particular biosynthesis of phenylpropanoids pathway between the TP and TX. Note: “TP” denotes the peel of *Gastrodiae Rhizoma*, while “TX” represents the core of *Gastrodiae Rhizoma*.

To investigate the mechanisms underlying the accumulation of DMs between the TP and TX, Kyoto Encyclopedia of Genes and Genomes (KEGG) pathway analysis was performed on the DMs, revealing enrichment in 10 metabolic pathways. A Sankey bubble plot highlighted the pathway of biosynthesis of phenylpropanoids as the primary divergent pathway between the TP and TX (Enrichment Ratio > 2, *p* < 0.05) (Figure 7, Supplementary Table S12). To visually illustrate changes in the levels of key network node metabolites between the most enriched metabolic pathways, we generated a metabolite-centric heatmap. Notably, metabolites such as formononetin, scopoletin, naringenin chalcone, isoliquiritigenin, and daidzein were primarily associated with phenylpropanoid biosynthesis. With the exception of scopoletin, all other metabolites were predominantly enriched in the TP. Furthermore, there were two flavonoids among these metabolites, which were consistent with our previous findings on total flavonoid content (Supplementary Figure S3).

### 2.4. Lifespan-Extending Potential Analysis

#### 2.4.1. Multiple-Concentration Screening of Test Concentration of the TP

**Figure 8:**
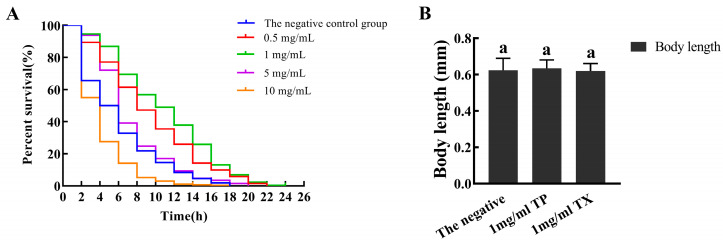
Multiple-concentration screening of test concentration of the TP ((**A**): Multiple-concentration screening of test concentration of the TP; (**B**): the body length of *C. elegans*). Note: 1. There is no significant difference between the same letters. 2. “TP” denotes the peel of *Gastrodiae Rhizoma*, while “TX” represents the core of *Gastrodiae Rhizoma*.

In the range of 0–5 mg/mL, the average lifespan of *C. elegans* in the TP group was significantly longer than that in the negative control group (*p* < 0.01, Table 3), with 1 mg/mL TP showing optimal efficacy. A high concentration of 10 mg/mL restrained the normal growth of *C. elegans* (Figure 8). Treatment with 1 mg/mL TP and TX showed no adverse effects on the body length of *C. elegans*, confirming the safety of the selected dosage. Therefore, the subsequent analysis of the lifespan-extending effects of the TP was conducted at a concentration of 1 mg/mL.

#### 2.4.2. Effects of the TP and TX on the Lifespan of *C. elegans*

**Figure 9:**
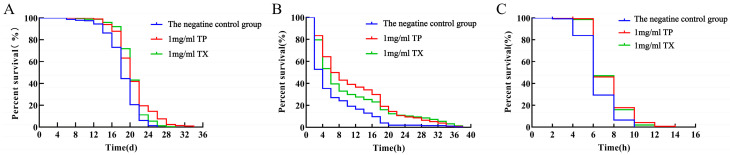
Effects of water extracts from different parts of *Gastrodiae Rhizoma* on the lifespan of *C. elegans* ((**A**): On the normal conditions; (**B**): On the resistance of *C. elegans* to oxidative stress; (**C**): On the resistance of *C. elegans* to heat stress). Note: “TP” denotes the peel of *Gastrodiae Rhizoma*, while “TX” represents the core of *Gastrodiae Rhizoma*.

Compared with the negative control group, the average lifespan of *C. elegans* in the 1 mg/mL TP group and 1 mg/mL TX group was prolonged by 13.98% and 11.77% (*p* < 0.01), and the maximum lifespan was prolonged by 8 d and 3.3 d. The longest lifespan of *C. elegans* in the 1 mg/mL TP group was significantly higher than the 1 mg/mL TX group and the negative control group (*p* < 0.01) (Table 4, Figure 9A).

Under 400 μM juglone-induced oxidative stress, *C. elegans* treated with 1 mg/mL TP showed a significantly longer mean lifespan (80.96%) than the negative control group (*p* < 0.01; Table 5, Figure 9B), while TX achieved a 61.03% lifespan extension. Under thermal stress at 37 °C, both 1 mg/mL TP and TX groups exhibited extended mean lifespans (13.94% and 15.33%, respectively; *p* < 0.01) compared to the negative control group (Table 6, Figure 9C). Collectively, TP and TX enhanced stress resistance in *C. elegans*, with no significant difference in lifespan extension between them (*p* > 0.05).

#### 2.4.3. Effects of the TP and TX on the Healthspan of *C. elegans*

**Figure 10:**
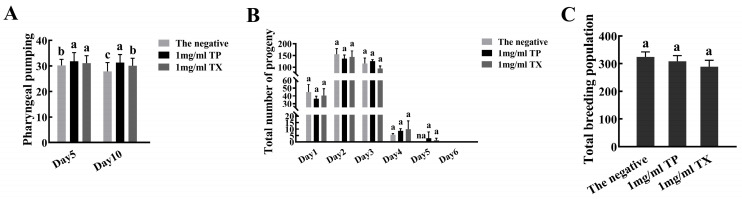
Effects of water extracts from different parts of *Gastrodiae Rhizoma* on the healthspan of *C. elegans* ((**A**): The pharyngeal pumping of *C. elegans*; (**B**,**C**): The reproductive capacity of *C. elegans*). Note: 1. The same letter in the Figure indicates that the difference is not significant, and different lowercase letters indicate a significant difference (*p* < 0.05). 2. “TP” denotes the peel of *Gastrodiae Rhizoma*, while “TX” represents the core of *Gastrodiae Rhizoma*. 3. The “na” represents that the data at this location is zero.

Compared with the negative control group, on the 5th and 10th days the 1 mg/mL TP group showed 5.71% and 12.18% increases in average pharyngeal pumping, while the TX group increased by 4.22% and 7.70%, respectively (*p* < 0.05) (Figure 10A). TP showed greater improvement in *C. elegans* pharyngeal pumping. The oviposition period of *C. elegans* in the administration group was one day longer than that of the negative control group, but there was no significant difference in the average daily oviposition and total oviposition (Figure 10B,C).

## 3. Discussion

The research on the compositional profiles and bioactivities of different plant parts holds significant implications for resource utilization. Pan et al. conducted comparisons of chemical characteristics and biological activities among various peony parts (root bark, root core, old stem, young stem, and leaves) using LC-MS-IT-TOF and multiple in vitro evaluation systems, demonstrating comparable phytochemical profiles and biological effects between the root core and the traditional medicinal root bark [43]. Zhao et al. discovered that both the peel and pulp of the wampee fruit demonstrate distinctive flavor profiles [44]. Although integrated multi-omics analysis has been widely applied to investigate chemical constituents in various plant tissues [39,45], its application in TP and TX remains undocumented in the literature. Our study compared TP and TX, revealing comparable phytochemical composition and lifespan-extending bioactivity. Notably, despite compositional similarities between TP and TX, the accumulation levels of specific compounds exhibited significant differences between them, highlighting a divergence in bioactive compound distribution.

The characteristic “horse-urine-like odor” serves as a crucial quality indicator for TM in traditional Chinese medicine practice, where stronger odor intensity correlates with superior medicinal quality [26]. The contribution of volatile compounds to sample odor profiles depends on their odor activity values (OAV) or ROAV [39]. ROAV analysis identified TP as the primary source of the characteristic aroma in TM, with key odorants including (*Z*)-4-heptenal, β-citronellol, hexanal, 1-pentanol, 1-octen-3-one, 2-methylpropanol, 3-methyl-2-butenal, 2-butanone, 2-methyl butanal, 3-methylthiopropanal, 3-penten-2-one, and 3-methyl butanal. Notably, 3-methylthiopropanal has been reported in prior studies as the pivotal contributor to TM’s unique fragrance [17,46], a finding consistent with the present study, which further revealed its predominant enrichment in TP. Among these compounds, β-citronellol, whose cardiovascular therapeutic efficacy (e.g., antihypertensive effects mediated through endothelium-independent vasodilation) has been pharmacologically validated [47,48], appears to underpin the empirical correlation between stronger TM aromas and enhanced medicinal potency. However, the “horse-urine-like odor” arises from synergistic interactions of multiple volatile components, and the pharmacological contributions of other odor-active constituents in TM warrant further investigation.

For metabolomic analyses, despite the samples originating from diverse geograpical locations and distinct TM formae, PCA revealed that all TP and all TX samples clustered distinctly into separate groups, demonstrating that the primary source of specialized metabolite variation stems from core-to-peel differentiation. This conclusion was further validated by hierarchical clustering heatmaps, where TP and TX were clustered into distinct categories respectively. Chemical constituents serve as the material basis for the pharmacological activities of medicinal agents. KEGG pathway analysis revealed that phenylpropanoid biosynthesis constitutes a critical differential metabolic pathway between TP and TX, with TP exhibiting significant enrichment of phenylpropanoids and polyketides. These compounds are associated with multifaceted bioactivities beneficial to human health: Formononetin demonstrated neuroprotective effects in a spinal cord injury model by reducing oxidative stress and inflammation, which helps regulate proteins involved in cell death and prevents neuronal damage [49]; Daidzein preserves mitochondrial integrity by blocking membrane potential collapse, caspase-3 activation, and NOX4-mediated oxidative stress, while ameliorating diabetic neuropathy [50,51]; and isoliquiritigenin enhances sciatic nerve NAD/NADH ratios to activate SIRT1, stimulate mitochondrial biogenesis, and alleviate diabetic neuropathy [52]. We hypothesize that the enrichment of these bioactive constituents in TP may contribute to its superior bioactivity.

The bioactivity of plant extracts typically arises from synergistic interactions among multiple constituents, necessitating holistic evaluation of raw materials for functional food development [53]. Given this context, we investigated the lifespan-extending potential of TP and TX using *C. elegans* model-based assays. Prior to lifespan assessment, toxicity assays of TP on *C. elegans* survival and body length were conducted. Toxicological studies suggest that tissue abnormalities correlate with genotoxic or teratogenic effects [36]. The results validated the appropriateness of the selected dosage for subsequent experiments (Figure 8). In our study, TP demonstrated comparable lifespan extension efficacy to TX in *C. elegans* (*p* < 0.01; Figure 9A, Table 3), providing preliminary evidence for TP’s potential anti-senescence activity. With aging, the pharyngeal pumping function of *C. elegans* gradually deteriorates, leading to reduced pumping activity [36,54]. Notably, longevity interventions may involve trade-offs between reproduction and lifespan [34]. To ensure functional health, we assessed feeding frequency and reproduction as healthspan indicators. TP maintained normal reproductive capacity (Figure 10C) and showed significantly improved pharyngeal pumping compared to the negative control and TX groups (*p* < 0.05; Figure 10A), indicating its superior healthspan-promoting potential over TX. This study investigated the lifespan-extending effects of TP and TX under thermal and oxidative stress, conditions known to accelerate cellular oxidation and drive aging processes [34,36]. We found that their lifespan-extending activity was likely mediated through enhanced antioxidant capacity in *C. elegans*, a mechanism corroborated by prior studies [54]. The correlation between lifespan extension and stress resistance in *C. elegans* has been previously documented. As outlined in the introduction, the antioxidant capacities of gastrodin, parishin, and adenosine derivatives have been experimentally validated. HPLC quantitative analyses revealed consistent concentration profiles of these bioactive constituents between TP and TX (Figure 6C,D), thus suggesting their potential role as a pharmacodynamic material basis underlying the observed lifespan-extending bioactivity. Furthermore, the pronounced enrichment of phenylpropanoids and polyketides in TP may constitute the material basis for its superior lifespan-extending potential compared to TX. However, the detailed pharmacological mechanisms warrant further validation through molecular-level investigations.

## 4. Materials and Methods

### 4.1. Materials and Reagents

Fresh TM was harvested in December 2022 and identified as the tuber of *Gastrodia elata* Blume by Professor Zhirong Sun of the School of Chinese Materia Medica at Beijing University of Chinese Medicine according to the morphological identification standards specified in the 2020 edition of the Chinese Pharmacopoeia [18]. To ensure the collected samples represent *Gastrodia elata* populations comprehensively, we established three sampling sites in its primary production areas: Moyugou Village, Long’an Town, Pingwu County, Sichuan Province; Maobahe Town, Ningqiang County, Shaanxi Province; Da’an Town, Ningqiang County, Shaanxi Province. Further, the TM samples comprise two distinct formae: *Gastrodia elata* BL. *f. elata* and *Gastrodia elata* BL. *f. glauca*. For subsequent analyses, samples will be selectively utilized based on specific research requirements. The specific sample table was shown in Supplementary Table S1. The wild-type *C. elegans* N2 and *Escherichia coli* OP50 were donated by the Institute of Genetics and Developmental Biology, Chinese Academy of Sciences. The mixed standard solution containing 2-butanone, 2-pentanone, 2-hexanone, 2-heptanone, 2-octanone, and 2-nonanone was purchased from Aladdin, Beijing, China (all analytically pure). The remaining reagents were purchased from Shanghai Yuanye Bio-Technology Co., Ltd. (Shanghai, China).

### 4.2. Preparation of the Whole TM, TP and TX

**Figure 11:**
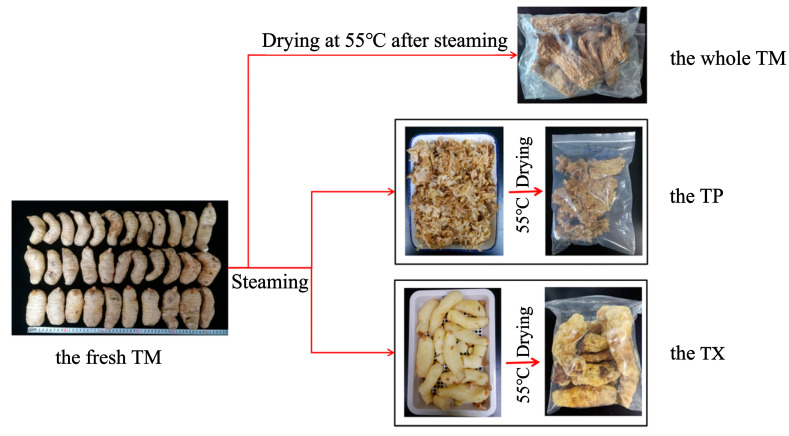
Processing flow chart of different parts of *Gastrodiae Rhizoma.* Note: TM: refers to the complete *Gastrodiae Rhizoma*. TP: refers to the 0–1.5 cm part under the epidermis of TM. TX: refers to the part of the whole TM after removing the TP.

The TM with weight in the range of 100–200 g was divided into three samples according to Figure 11. Fresh TM was cleaned with tap water and was air-dried at room temperature until surface moisture evaporated, then steamed to ensure no white core inside. Then, it was placed at room temperature, cooled slightly, and a portion was transferred to an oven and dried at 55 °C to constant weight, crushed, passed through the 65-mesh sieve and reserved for use [22]. The other portion was separated into TP and TX using forceps, with TP thickness maintained at 0–1.5 cm. After being baked at 55 °C until reaching constant weight, the samples in each group were crushed separately and passed through the 65-mesh sieve for subsequent use.

### 4.3. HPLC Analysis

#### 4.3.1. Chromatographic Conditions

The HPLC analysis was conducted using an LC-20AT high-performance liquid chromatograph (Shimadzu, Kyoto, Japan), and the elution conditions were modified based on a previous report [22]. The Diamonsil C18 chromatographic column (250 mm × 4.6 mm, 5 μm) was employed, with column temperature maintained at 35 °C, detection wavelength set at 270 nm, injection volume of 15 μL, and flow rate of 1.5 mL/min. The mobile phase was composed of 0.1% formic acid (A) and acetonitrile (B). The gradient elution conditions were as follows: 0–5 min, 1% B; 5–15 min, 1–2% B; 15–25 min, 2–8% B; 25–35 min, 8% B; 35–45 min, 8–15% B; 45–55 min, 15% B.

#### 4.3.2. Fingerprints Similarity Evaluation

The 2012 edition of ‘Traditional Chinese Medicine Fingerprint Similarity Evaluation System’ software (v 2012), conventionally employed to assess quality variations among different batches or geographical origins of herbal medicines [55,56], was adapted in this study to analyze the differences between TP, TX, and the whole TM. After importing HPLC fingerprints of the samples into the software, parameters were initially set according to the method of Lv et al. with further optimization [56], specifically employing the median method with a time window width of 0.2 min. Following multipoint correction, automatic peak matching was conducted.

#### 4.3.3. Standard Solution Preparation

A mixed reference solution was prepared by dissolving accurately weighed amounts of adenosine (0.096 mg/mL), gastrodin (1.188 mg/mL), p-hydroxybenzyl alcohol (0.89 mg/mL), parishin E (0.89 mg/mL), parishin B (1.358 mg/mL), parishin C (0.564 mg/mL), and parishin A (2.032 mg/mL) in distilled water. The solution was diluted to prepare a series of concentration reference solutions using distilled water, and linear regression equations were established. The calculation of the LOD and LOQ were done for these ingredients using the standard deviation of the residuals from the calibration curve regression analysis (Supplementary Table S2).

#### 4.3.4. Sample Preparation

Powder samples of both TP and TX (2 g each) were ultrasonically extracted with 50 mL of 70% ethanol (500 W, 40 kHz) for 30 min. After cooling and centrifugation (4000 r/min, 5 min), 20 mL of supernatant was collected, evaporated to dryness, and reconstituted in 10% acetonitrile to a final volume of 5 mL. The solution was filtered through a 0.22-μm membrane prior to analysis.

#### 4.3.5. Qualitative and Quantitative Analyses

Peaks were identified and quantified by comparing their retention times and areas with reference standards, using the established calibration curves.

### 4.4. GC–IMS Analysis

The GC–IMS FlavourSpec^®^ instrument (G.A.S. Dortmund, Dortmund, Germany) was used for sample analysis. Samples TP1 and TX1 from Supplementary Table S1 were selected for analysis. An amount of 0.1 g of TM powder was taken into a sealed 20 mL headspace vial. The headspace injection conditions were incubated at 80 °C for 20 min. After incubation, 500 μL was injected at 85 °C (splitless injection). Then, the samples were driven into MXT-5 capillary column (15 m × 0.53 mm, 1.0 μm) (60 °C isothermal conditions) by nitrogen (purity ≥ 99.999%) at a programmed flow as follows: 2.0 mL/min for 2 min, linearly increasing to 10.0 mL/min over 8 min, 100.0 mL/min over 10 min, 150.0 mL/min over 10 min, and the chromatographic run time was 30 min. The conditions of IMS were as follows: the migratory tube temperature was set to 45 °C, and the flow rate was maintained at 75 mL/min with high-purity nitrogen (purity ≥ 99.999%) as the drift gas (positive ion mode). All analyses were performed in triplicate. 2-butanone, 2-pentanone, 2-hexanone, 2-heptanone, 2-octanone, and 2-nonanone (all analytically pure, purchased from Aladdin, Beijing, China) were used to prepare standard mixes. Then, the mixing of six ketones was detected, the calibration curve of retention time and retention index was established, and then the retention index of the substance was calculated from the retention time of the target, and the qualitative analysis of the target was carried out using the built-in GC retention index (NIST 2020) database and IMS migration time database of VOCal software (0.4.03) (G.A.S. Dortmund, Germany). The relative concentration (%) of volatile compounds was calculated using Equation (1), where ***A_i_*** represents the peak volume of the target compound.

(1) $$Celative\;Concentration\;(\%)=\frac{A_{i}}{\sum A_{i}}\times 100\%$$

The contribution of each compound to the overall flavor profile was assessed via relative odor activity value (ROAV), with key odorants identified as those exhibiting ROAV ≥ 1. For ROAV analysis, only relative concentration data were required [39]. ROAV were determined via Equation (2), where ***C_i_*** and ***T_i_*** denote the concentration and the threshold value of the compound, respectively, and ***T_max_***/***C_max_*** represents the maximum ***T_i_***/***C_i_*** ratio among all the compounds.

(2) $$ROAV=\frac{C_{i}}{T_{i}}\times \frac{T_{max}}{C_{max}}\times 100$$

### 4.5. LC-MS Analysis

A total of 100 mg of liquid nitrogen-ground samples were added with 500 μL of 80% methanol aqueous solution, vortexed and shaken, placed in an ice bath for 5 min, and centrifuged at 15,000× *g* at 4 °C for 20 min. The sample identifications correspond to the codes in the Supplementary Table S1. A certain amount of supernatant was diluted with mass spectrometry-grade water to 53% methanol content, centrifuged at 15,000× *g* and 4 °C for 20 min, and the supernatant was collected for injection analysis. Then, the samples were analyzed using the Vanquish ultra-high performance liquid chromatography system (UHPLC, Thermo Fisher, Waltham, MA, USA). The chromatographic column used was a Hypersil Gold column (C18) (100 mm × 2.1 mm, 1.9 μm) (Thermo Fisher, Waltham, MA, USA). Data acquisition was performed separately in positive and negative ion modes. For positive ionization mode, the mobile phase was composed of mobile phase A: 0.1% formic acid, and B: methanol. For negative ionization mode, the mobile phase consisted of A: 5 mM ammonium acetate, pH 8.0, and mobile phase B: methanol. The flow rate was 0.2 mL/min, and column temperature was set at 40 °C. The gradient elution procedure was as follows: 0–1.5 min, 2% B; 1.5–3 min, 2–85% B; 3–10 min, 85–100% B; 10–10.1 min, 100–2% B; 10.1–11 min, 2% B; 11–12 min, 2% B. The mass spectrometry was conducted by Q Exactive HF-X (Thermo Fisher, USA). Spray voltage: 3.5 kV; sheath gas flow rate: 35 psi; auxiliary gas flow rate: 10 L/min; ion transport tube temperature: 320 °C; ion transfer RF level: 60; auxiliary gas heater temperature: 350 °C; MS/MS scans were performed in data-dependent mode; scanning range: *m*/*z* 100–1500. The CD3.1 software (Thermo Fisher, USA) was used to process the original LC-MS data. The retention time deviation of 0.2 min and the mass deviation of 5 ppm were set for peak alignment. The mass deviation of 5 ppm and the signal intensity deviation of 30% were used for peak extraction.

Following raw data preprocessing, molecular feature identification was performed by matching against a high-quality mzCloud database constructed from authentic standards (Level 1 identifications) alongside the mzVault and MassList databases (Level 2 annotations) [17,57,58]. The identification principles were applied as follows: Molecular weight determination was based on precursor ion *m*/*z* from primary MS, followed by molecular formula prediction using mass accuracy (ppm) and adduct information prior to database matching; for databases containing MS/MS spectra, secondary identification was achieved by matching experimental fragment ion patterns and collision energy parameters against reference spectral libraries. Metabolites exhibiting a coefficient of variance (CV) < 30% [59] in QC samples were subsequently retained as definitive identification results for downstream analysis.

### 4.6. Lifespan-Extending Potential of Different Parts of TM in C. elegans

#### 4.6.1. Preparation of Different Parts of TM Water Extract Administration Extract Solution

Samples TP1 and TX1 from Supplementary Table S1 were selected for analysis. An appropriate amount of TP and TX powder was accurately weighed and placed in a conical flask with a stopper, respectively. Distilled water was added at a solid–liquid ratio of 1: 20, refluxed in a hot water bath for 1.5 h, and centrifuged at 4000 r/min for 10 min. The supernatant was collected and extracted once more, concentrated by rotary evaporation at 40 °C, and vacuum freeze-dried to obtain a freeze-dried powder of the extract, which was stored in a refrigerator at −20 °C for standby.

#### 4.6.2. Culture of *E. coli* OP50 and *C. elegans*

*E. coli* OP50 mother liquor (10 μL) was added to 50 mL of LB liquid medium, followed by shaking culture at 37 °C for 24 h. The culture was then stored at 4 °C for ≤ 15 d. The N2 *C. elegans* were maintained at 20 °C on nematode growth medium (NGM) agar plates containing *E. coli* OP50. To ensure developmental synchrony, L4-stage *C. elegans* were obtained through synchronization method for subsequent experimental procedures. The pyrolysis solution was prepared as follows: 0.2 mL of 5 M NaOH, 0.8 mL of 5% NaOCl, and 4 mL of M9 solution were fully mixed and freshly prepared [60,61].

#### 4.6.3. Multiple-Concentration Screening of the Drug Group and Design of Experimental Groups

The appropriate amount of freeze-dried powder of the extract was dissolved in sterile water, passed through a 0.22 μm sterile filter, and mixed with the bacterial solution to prepare a bacterial solution with a certain concentration of the drug. The same volume of sterile water was mixed with the bacterial solution to prepare a negative control group bacterial solution. The *C. elegans* model was used to assess its lifespan-extending effects.

Given that the primary objective of this study is to analyze the potential application value of TP, we evaluated the effects of TP extracts (0–10 mg/mL) on the lifespan of *C. elegans* under oxidative stress conditions, aiming to identify the optimal concentration for subsequent lifespan-extending research.

#### 4.6.4. Measurements of Body Length

*C. elegans* were cultured as described in Section 4.6.2. On the 10th day of administration, the *C. elegans* were washed with M9, and the anesthetics were added. After the worms’ bodies were stiff, they were observed and photographed on an inverted fluorescence microscope, and the body length was measured by ImageJ software (1.54a). The experiment was repeated three times independently.

#### 4.6.5. Lifespan Assay

The synchronized N2 *C. elegans* were transferred to the negative control group and the administration group, thirty in each plate, three plates in each group, and the plates were rotated every 2 d. The survival of the *C. elegans* was observed. If the body of the *C. elegans* was repeatedly touched and did not react, the worm was regarded as dead.

#### 4.6.6. Reproduction Assays

There were five plates in each group, two *C. elegans* in each plate, and the plates were rotated at an interval of 24 h until the *C. elegans* no longer laid eggs. Finally, the hatched nematodes were counted. The experiment was repeated three times independently.

#### 4.6.7. Pharyngeal Pumping

*C. elegans* were cultured as described in Section 4.6.2. On the 5th and 10th day of administration, the pharyngeal pumping of *C. elegans* during a 20-s observation period was recorded under a stereomicroscope, and the number of pumps of the *C. elegans* was counted. A total of thirty *C. elegans* were counted in each group. The experiment was repeated three times independently.

#### 4.6.8. Heat Stress and Oxidative Stress Tests

*C. elegans* were cultured as described in Section 4.6.2. After 5–6 d of administration, for heat stress, the incubator temperature was adjusted to 37 °C for survival monitoring; for oxidative stress, the *C. elegans* were transferred to NGM plates containing 400 μM juglone. The survival of *C. elegans* was observed every 2 h until all *C. elegans* died. The experiment was repeated three times independently.

### 4.7. Statistical Analysis

IBM SPSS Statistics V.27.0.1 was used for statistical analysis and GraphPad.Prism.9.5 was used for generating figures. All experiments were performed a minimum of three times. Statistical comparisons were made using one-way analysis of variance (ANOVA) and Student’s *t*-test. The assumptions of normality and homogeneity of variance were verified using the Shapiro–Wilk test and Levene’s test, respectively. Multivariate statistical analyses were performed using MetaboAnalyst 6.0 (https://new.metaboanalyst.ca/, accessed on 17 March 2025), including principal component analysis (PCA) and orthogonal partial least squares–discriminant analysis (OPLS–DA). Prior to PCA and OPLS–DA, raw data were normalized using Pareto scaling. The OPLS–DA model underwent rigorous validation through leave-one-out cross-validation and permutation testing. Heatmap analysis was performed using the ComplexHeatmap package (v2.20.0) in R (v4.4.1), with metabolite intensities standardized via Z-score transformation before analysis. KEGG pathway enrichment was conducted using the clusterProfiler package (v4.12.0) in R (v4.4.1), wherein metabolites were first annotated by the Kyoto Encyclopedia of Genes and Genomes (KEGG, https://www.kegg.jp/kegg/, accessed on 1 April 2025).

## 5. Conclusions

This study elucidated the differences in chemical profiles and lifespan-extending potential between TP and TX through an integrated analytical framework combining GC–IMS, UHPLC–MS, HPLC, and *C. elegans* models. The distinctive odor of TM is positively correlated with its quality. The integrated analysis employing GC–IMS technology combined with ROAV ≥ 1 demonstrated that TP plays a primary role in constituting the characteristic odor profile of TM. Characteristic odor-active compounds (twelve in total) were identified in TP and TX, with seven components exhibiting significant enrichment specifically in TP, including (*Z*)-4-heptenal, β-citronellol, hexanal, 1-pentanol, 1-octen-3-one, 2-methylpropanol, and 2-butanone. However, the structure–activity relationships between key odorants and traditional pharmacological efficacy require further confirmation via targeted validation assays. Untargeted metabolomics provided metabolic-level insights into their biological distinctions, revealing that TP-TX divergence predominantly localizes to phenylpropanoid biosynthesis pathways. In lifespan-extending potential, TP exhibited comparable lifespan extension to TX in *C. elegans* but demonstrated superior improvement in pharyngeal pumping function, potentially linked to enhanced antioxidant capacity. This bioactivity may correlate with TP’s comparable levels of gastrodin derivatives, parishins, and adenosine to TX, coupled with its significant phenylpropanoid enrichment. Future investigations would benefit from integrating in vitro enzyme activity assays, validated molecular docking, and multi-omics strategies to quantitatively elucidate structure–activity relationships and underlying signaling pathways. In conclusion, our findings suggest that retaining TP during TM processing is superior to removing it, as TP preservation may better maintain the overall distinctive aroma profile of TM while retaining more bioactive components. This discovery provides a novel scientific rationale for TM utilization in food and pharmaceutical industries.

## Figures and Tables

**Table 1:** Similarity of HPLC chromatograms of *Gastrodiae Rhizoma* and its peel and core.

Number	Similarity	Number	Similarity	Number	Similarity
TM1	0.967	TP1	0.972	TX1	0.959
TM2	0.971	TP2	0.971	TX2	0.967
TM3	0.98	TP3	0.969	TX3	0.889
TM4	0.984	TP4	0.867	TX4	0.974

Note: “TM” refers to the whole of *Gastrodiae Rhizoma*, “TP” denotes the peel of *Gastrodiae Rhizoma*, and “TX” represents the core of *Gastrodiae Rhizoma*.

**Table 2:** The relative odor activity values (ROAV) for different volatile compounds of TX and TP.

Compound	Threshold Odor (μg/L)	ROAV	
		TX	TP
(*Z*)-4-heptenal	0.0034	43.622	100
Beta-Citronellol	0.01	100	53.939
Hexanal	0.23	46.259	42.304
1-Pentanol	0.153	19.722	28.57
1-Octen-3-one	0.016	14.838	11.881
2-methylpropanol	0.33	1.495	9.959
3-Methyl-2-butenal	0.5	<1	1.916
2-Butanone	1.3	1.926	1.915
2-Methyl butanal	5.2	<1	1.861
3-Methylthiopropanal	0.43	<1	1.359
3-Penten-2-one	1.2	<1	1.339
3-Methyl butanal	5.4	<1	1.061

Note: 1. Odor threshold from the literature: [37,38,39,40,41,42]. 2. “<1” indicates that the ROAV of this compound was less than 1, and was not main typical odor compound of the sample. 3. “TP” denotes the peel of *Gastrodiae Rhizoma*, while “TX” represents the core of *Gastrodiae Rhizoma*.

**Table 3:** Effects of water extracts from the TP on the lifespan of *C. elegans* under oxidative stress (x ± s, n = 3).

Group	Average Life/h	Median Life/h	Longest Life/h
The negative control group	6.066 ± 0.261 ^D^	6	16.667 ± 2.309 ^A^
0.5 mg/mL	9.523 ± 0.323 ^B^	8	20.667 ± 1.155 ^A^
1 mg/mL	10.950 ± 0.328 ^A^	10	22.000 ± 2.000 ^A^
5 mg/mL	8.344 ± 0.289 ^C^	6	18.667 ± 4.163 ^A^
10 mg/mL	4.148 ± 0.166 ^E^	4	14.667 ± 4.163 ^A^

Note: 1. There is no significant difference between the same letters of the same column in the table, and the difference between the different uppercase letters is highly significant (*p* < 0.01). 2. “TP” denotes the peel of *Gastrodiae Rhizoma*.

**Table 4:** Effects of water extracts from different parts of *Gastrodiae Rhizoma* on the lifespan of *C. elegans* (x ± s, n = 3).

Group	Average Life/h	Median Life/h	Longest Life/h
The negative control group	18.317 ± 0.934 ^B^	18	25.333 ± 1.155 ^Bc^
1 mg/mL the TP group	20.878 ± 0.201 ^A^	20	33.333 ± 1.155 ^Aa^
1 mg/mL the TX group	20.472 ± 0.347 ^A^	20	28.667 ± 1.155 ^Bb^

Note: 1. The same letter in the table indicates no significant difference, different lowercase letters indicate a significant difference (*p* < 0.05), and different uppercase letters indicate a highly significant difference (*p* < 0.01). 2. “TP” denotes the peel of *Gastrodiae Rhizoma*, while “TX” represents the core of *Gastrodiae Rhizoma*.

**Table 5:** Effects of water extracts from different parts of *Gastrodiae Rhizoma* on the lifespan of *C. elegans* under oxidative stress (x ± s, n = 3).

Group	Average Life/h	Median Life/h	Longest Life/h
The negative control group	6.451 ± 0.415 ^B^	4	36.670 ± 1.155 ^A^
1 mg/mL the TP group	11.674 ± 0.602 ^A^	8	36.670 ± 2.309 ^A^
1 mg/mL the TX group	10.388 ± 0.659 ^A^	6	37.330 ± 1.155 ^A^

Note: 1. There is no significant difference between the same letters of the same column in the table, and the difference between the different uppercase letters is highly significant (*p* < 0.01). 2. “TP” denotes the peel of *Gastrodiae Rhizoma*, while “TX” represents the core of *Gastrodiae Rhizoma*.

**Table 6:** Effects of water extracts from different parts of *Gastrodiae Rhizoma* on the lifespan of *C. elegans* under heat stress.

Group	Average Life/h	Median Life/h	Longest Life/h
The negative control group	6.378 ± 0.098 ^B^	6	10.000 ± 0.000 ^b^
1 mg/mL the TP group	7.267 ± 0.099 ^A^	6	11.333 ± 1.1547 ^ab^
1 mg/mL the TX group	7.356 ± 0.109 ^A^	6	12.667 ± 1.1547 ^a^

Note: 1. The same letter in the table indicates no significant difference, different lowercase letters indicate a significant difference (*p* < 0.05), and different uppercase letters indicate a highly significant difference (*p* < 0.01). 2. “TP” denotes the peel of *Gastrodiae Rhizoma*, while “TX” represents the core of *Gastrodiae Rhizoma*.

## Data Availability

The data are included in this article.

## Supplementary Materials

The following supporting information can be downloaded at: https://www.mdpi.com/article/10.3390/molecules30173474/s1, refs. [62,63] are cited in Supplementary Materials.
