# Supplementary material for: Comparative Phytochemical Analysis of Gastrodiae Rhizoma Peel and Core and Their Lifespan-Extending Potential in Caenorhabditis elegans
Source: Molecules. 2025 Aug 23;30(17):3474. doi: 10.3390/molecules30173474 (PMC12430696; doi:10.3390/molecules30173474)
Supplement: Supplementary file 1 [file molecules-30-03474-s001.zip › Supplementary Methods.docx]

**Detection of total flavonoids and total polyphenols content**

Following established protocols[1], precisely weighed 2.0 g of TP and TR powder was subjected to aqueous extraction using a 1:25 (w/v) solid-to-solvent ratio, then homogenization twice (12 000 r/min, 2min). And centrifuged again (4 000 r/min, 20min) after ultrasonic (550 W, 40 kHz, 60 min). The clarified supernatant was volumetrically adjusted to 25 mL with ultrapure water for content detection. The rutin standard solution with the final concentration of 5, 15, 30, 60, 120, 150, 180 and 200 μg/mL was prepared, and the absorbance of the standard solution was determined. The regression equation was Y = 0.0052X + 0.0073 (R^2^ = 0.9998). The sample extract was used to determine the content of total flavonoids, expressed as mg RE / g. Gallic acid standard solutions with final concentrations of 5, 25, 50, 75, 100, 150, 200, 250 μg/mL were prepared, and the absorbance of the standard solution was measured. The regression equation was Y = 0.0041X-0.0012 (R^2^ = 0.9990). The sample extract was used to determine the total polyphenol content, expressed as mg GAE / g.

**References**

1. Liao;, X.; Wu;, Z.; Yang;, Y.; Chen;, G.; Zhan, Y. The differentiation of total polyphenols and total flavonoids contents in Gastrodia elata Bl. f. glauca S. Chow extracts using different solvents and their antioxidant capacities. *Food and Fermentation Industries* **2022**, *5*, 8, doi:10.13995/j.cnki.11-1802/ts.027943.
