# Supplementary material for: Comparative Phytochemical Analysis of Gastrodiae Rhizoma Peel and Core and Their Lifespan-Extending Potential in Caenorhabditis elegans
Source: Molecules. 2025 Aug 23;30(17):3474. doi: 10.3390/molecules30173474 (PMC12430696; doi:10.3390/molecules30173474)
Supplement: Supplementary file 1 [file molecules-30-03474-s001.zip › Supplementary Table S1.docx]

**Table S1 Sample tables of different parts of** *Gastrodiae Rhizoma*

| **number** | formae | **sampling site** | **harvest time** | phenologycal status | longitude | latitude |
| --- | --- | --- | --- | --- | --- | --- |
| **H1** | ***G. elata* BL. *f. elata*** | **Da 'an Town, Ningqiang County, Shaanxi Province** | **2022.12.15** | dormancy period | 106.30°E | 33.06°N |
| **W1** | ***G. elata* BL. *f. glauca*** | **Maobahe Town, Ningqiang County, Shaanxi Province** | **2022.12.26** | dormancy period | 106.34°E | 32.68°N |
| **H2** | ***G. elata* BL. *f. elata*** | **Moyugou Village, Long 'an Town, Pingwu County, Sichuan Province** | **2022.12.6** | dormancy period | 104.56°E | 32.41°N |
| **W2** | ***G. elata* BL. *f. glauca*** | **Moyugou Village, Long 'an Town, Pingwu County, Sichuan Province** | **2022.12.6** | dormancy period | 104.56°E | 32.41°N |
| **TP1** | ***G. elata* BL. *f. elata*** | **Da 'an Town, Ningqiang County, Shaanxi Province** | **2022.12.15** | dormancy period | 106.30°E | 33.06°N |
| **TP2** | ***G. elata* BL. *f. glauca*** | **Maobahe Town, Ningqiang County, Shaanxi Province** | **2022.12.26** | dormancy period | 106.34°E | 32.68°N |
| **TP3** | ***G. elata* BL. *f. elata*** | **Moyugou Village, Long 'an Town, Pingwu County, Sichuan Province** | **2022.12.6** | dormancy period | 104.56°E | 32.41°N |
| **TP4** | ***G. elata* BL. *f. glauca*** | **Moyugou Village, Long 'an Town, Pingwu County, Sichuan Province** | **2022.12.6** | dormancy period | 104.56°E | 32.41°N |
| **TR1** | ***G. elata* BL. *f. elata*** | **Da 'an Town, Ningqiang County, Shaanxi Province** | **2022.12.15** | dormancy period | 106.30°E | 33.06°N |
| **TR2** | ***G. elata* BL. *f. glauca*** | **Maobahe Town, Ningqiang County, Shaanxi Province** | **2022.12.26** | dormancy period | 106.34°E | 32.68°N |
| **TR3** | ***G. elata* BL. *f. elata*** | **Moyugou Village, Long 'an Town, Pingwu County, Sichuan Province** | **2022.12.6** | dormancy period | 104.56°E | 32.41°N |
| **TR4** | ***G. elata* BL. *f. glauca*** | **Moyugou Village, Long 'an Town, Pingwu County, Sichuan Province** | **2022.12.6** | dormancy period | 104.56°E | 32.41°N |

**注：1.** *Gastrodia elata* BL. (*G. elata* BL); *Gastrodiae Rhizoma* (TM).

2. To ensure the generalizability of conclusions, different formae of TM from various regions were grouped together.

3. The optimal harvest period for TM is during its dormancy period (Yang; et al., 2015); however, the timing varies due to varying climatic conditions across regions.

References

1. **Yang, X., Li, Y., Luo Y., Yu G., Zuo Y., & Pei, S. The Normalize Harvest Process and Classification of the Gastrodia elata. *Forest By-Product and Speciality in China* **2015**, *4, 47-48*,** <https://doi.org/10.13268/j.cnki.fbsic.2015.04.016>**.**
