# Supplementary material for: Comparative Phytochemical Analysis of Gastrodiae Rhizoma Peel and Core and Their Lifespan-Extending Potential in Caenorhabditis elegans
Source: Molecules. 2025 Aug 23;30(17):3474. doi: 10.3390/molecules30173474 (PMC12430696; doi:10.3390/molecules30173474)
Supplement: Supplementary file 1 [file molecules-30-03474-s001.zip › Supplementary Table S2.docx]

**Table 2 Standard curve linear regression equation ( n = 3 )**

| **active ingredient** | **equation of linear regression** | **R^2^** | **range of linearity（mg/mL）** | **LOD (mg/mL)** | **LOQ (mg/mL)** |
| --- | --- | --- | --- | --- | --- |
| adenosine | **y=20211528.7846x-7596.1846** | **0.9997** | **0.096~0.008268** | **0.002728** | **0.008268** |
| gastrodin | **y=1529970.7272x-12222.8143** | **0.9998** | **1.188~0.095587** | **0.031544** | **0.095587** |
| p-hydroxybenzyl alcohol | **y=5418726.5026x+59927.0770** | **0.9991** | **0.89~0.115421** | **0.038089** | **0.115421** |
| Parishin **E** | **y=691859x+4942.2** | **0.9996** | **0.89~0.079478** | **0.026228** | **0.079478** |
| Parishin **B** | **y=927043x+13156** | **0.9992** | **1.358~0.156203** | **0.051547** | **0.156203** |
| Parishin **C** | **y=979992x+6545.9** | **0.9993** | **0.564~0.063314** | **0.020894** | **0.063314** |
| Parishin **A** | **y=1049611.9971x+18647.4010** | **0.9995** | **2.032~0.186736** | **0.061623** | **0.186736** |

**Note:** 1. The limit of detection (LOD) was calculated using Equation (1), while the limit of quantitation (LOQ) was determined using Equation (2). In these equations, k represents the slope. The standard error of the estimate (Se) required for Equation (3) was calculated using Equation (3), where n denotes the number of data points used for linear fitting, y is the measured response value corresponding to the concentration, and y' is the fitted response value.

$$\begin{aligned} LOD=3.3\times\frac{S_{e}}{k}\#\left( 1 \right) \end{aligned}$$

$$\begin{aligned} LOQ=10\times\frac{S_{e}}{k}\#\left( 2 \right) \end{aligned}$$

$$\begin{aligned} S_{e}=\sqrt{\frac{\sum_{i-1}^{n} \left( y_{i}-y_{i}^{'} \right)^{2}}{n-2}}\#\left( 3 \right) \end{aligned}$$

2. The lower limit of the linear range is the LOQ (Limit of Quantitation).
